# Research Progress and Hotspots Analysis of Apoplastic Barriers in the Roots of Plants Based on Bibliometrics from 2003 to 2023

**DOI:** 10.3390/plants13233285

**Published:** 2024-11-22

**Authors:** Chongyuan Qin, Ruoqi Li, Zhuoran Tan, Jingnan Zhang, Yuyang Sun, Jinji Han, Xiaoxia Deng, Fei Wang, Qingjie Yang, Jinghong Wang, Jixiang Lin

**Affiliations:** 1 College of Landscape Architecture, Northeast Forestry University, Harbin 150040, China; 2 Social Science & Public Policy, School of Global Affairs, King’s College London, London WC2R 2LS, UK

**Keywords:** apoplastic barriers, casparian strip, suberin lamellae, bibliometrics, CiteSpace

## Abstract

The apoplastic barriers, composed of Casparian strip (CS) and suberin lamellae (SL), are integral to the regulation of water and plant nutrient uptake in plants, as well as their resilience to abiotic stresses. This study systematically examines the research developments and emerging trends in this field from 2003 to 2023, utilizing bibliometric tools such as Web of Science, CiteSpace, and VOSviewer to analyze a dataset of 642 publications. This paper reviews the cooperation of different countries, institutions, and scholars in apoplastic barriers research based on cooperative network analysis. In the field, China has the highest number of publications, the University of Bolton has the highest number of publications, and Niko Geldner is the author with the maximum number of publications. Notably, 27 publications were identified as highly cited, with their research primarily focusing on (1) genes, proteins, enzymes, and hormones regulating the formation of apoplastic barriers; (2) the influence of adversity stress on apoplastic barriers; (3) the chemical components of apoplastic barriers; (4) the evaluations of research progress on apoplastic barriers. Combined with the keyword co-occurrence network diagram, it is proposed that future research directions in this field should be as follows: (1) physiological functions of apoplastic barriers in plant root; (2) differences in the formation of apoplastic barriers with different root systems; (3) methods to promote apoplastic barriers formation; and (4) application of molecular biology techniques. The present study provides a further understanding of the trends in apoplastic barriers, and the data analyzed can be used as a guide for future research directions.

## 1. Introduction

As sessile organisms, the primary function of a plant’s root system is to absorb water and minerals and transport them to the above-ground parts. The water and plant nutrients are radially transported from the rhizosphere to the root center via three different pathways: (1) transcellular, (2) symplastic, and (3) apoplastic [1]. The first two pathways are also referred to as cell-to-cell pathways. The apoplastic pathway can be blocked by a Casparian strip (CS, also called Casparian band) and suberin lamellae (SL) in endodermal and exodermal cell walls [2]. A recent report has demonstrated that the formation of apoplastic barriers is an essential evolutionary adaptation of seed plants to the terrestrial environment [3].

The ability of apoplastic barriers to block water and nutrient movement is determined by their chemical composition. Suberin is a heterogeneous biopolymer composed of aliphatic monomers and aromatic moieties. The aliphatic monomers include primary alcohols, fatty acids, α–ω dicarboxylic acids (diacids), and ω-hydroxy acids (ω-OH acids), whereas the aromatic components include ferulic and coumaric acids. The content of different components content varies according to the species and environmental conditions [4]. The main chemical constituent of the CS is lignin. Whether the CS contains suberin is controversial. In 2012, Naseer et al. found by histochemical analysis and staining that early-stage CS in the roots of *Arabidopsis thaliana* contained only lignin, whereas mature CS contained both lignin and suberin [5]. Fourier-transform infrared spectroscopy and stimulated Raman scattering were applied to analyze the composition of CS in maize (*Zea mays*). The results showed that both lignin and suberin were present in the CS of maize roots [6], and the two components were deposited almost simultaneously. Similar results were found in China fir (*Cunninghamia lanceolata*) [7]. This variation implied that the chemical composition of CS might be related to the species and different developmental stages.

Both abiotic and biotic stresses have been demonstrated to promote the formation of apoplastic barriers, and the plasticity is highly genotype-dependent [8]. For example, cadmium (Cd) stress can induce the deposition of apoplastic barriers in the roots of wheat (*Triticum aestivum*), which increases under the high intensity of the stress [9]. In addition, low Cd stress delays the formation of apoplastic barriers, but higher Cd stress promotes the formation of apoplastic barriers in *Sedum alfredii* [10]. Some reports also indicate that the initiation of CS occurs earlier in the Na^+^-tolerant rice (*Oryza sativa*) cultivars than in Na^+^-sensitive rice cultivars, irrespective of the Na^+^ concentration [11]. The apoplastic barriers play a crucial role in nutrient retention and the exclusion of toxic substances in crops, making them valuable targets in crop breeding programs [12].

Recent reviews have focused on various aspects of apoplastic barriers, including prospects for abiotic stress tolerance [13], developmental mechanisms of CS [14], and the regulation of suberin formation [15], have been conducted by researchers in this field. However, studies examining the progress and hotspots of the entire field of research from a macro perspective are still scarce, especially in terms of the evolution of research trends and future development. Bibliometrics is a comprehensive knowledge system that integrates mathematics, statistics, and philology, focusing on quantification [16]. This study analyzed the literature on root apoplastic barriers in the Web of Science using CiteSpace [17] and VOSviewer [18]. We aimed to understand (1) publication trends on root apoplastic barriers and the collaboration of scholars with others; (2) highly cited publications and the reason for their citation; and (3) evolving research hotspots in the root apoplastic barriers. The study aimed to help researchers understand current research dynamics and hotspots deeply and to provide data reference and a basis for the in-depth exploration of root apoplastic barriers.

## 2. Data and Methodology

### 2.1. Data Sources

The online literature was searched through the Science Citation Index Expanded of the Web of Science Core Collection (WoS, Clarivate Analytics) on 31 December 2023. This database was selected because of its high quality and rich literature coverage, and further, it was highly suitable for mainstream bibliometric software [19]. The retrieval formula for this subject was TS = (apoplastic barriers OR Casparian strip OR Casparian band OR suberin lamellae). We manually excluded publications that did not fit the purpose of this study, such as those investigating the CS response of plant stems to stress. Overall, we downloaded 642 publications, including 512 research articles, 89 reviews, and 41 editorials.

### 2.2. Analysis Methods

Bibliometric analysis and visualization were performed using CiteSpace (version 6.2) and VOS viewer (version 1.6). Multiple units extracted from the dataset, including journal, author, institution, country, and keyword, were used to conduct the bibliometric analysis.

## 3. Results and Discussion

### 3.1. Bibliometric and Cooperation Network Analysis

**Figure 1:**
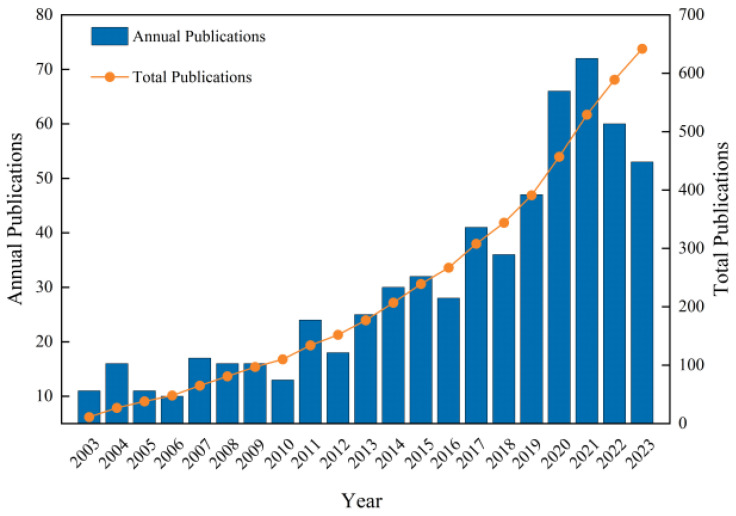
Annual and cumulative publication trends on apoplastic barriers from 2003 to 2023.

The results of the statistical analysis of the number of publications on apoplastic barriers obtained from the WoS database are shown in Figure 1. The number of publications published annually in this sector has increased. The highest number of publications in 2021 was 72, which was 7.2 times higher than that in 2006. From 2003 to 2010, the number of annual publications in this field was less than 20, which mainly analyzed the root systems of different plants to understand the differences in CS in various plant species. Some publications also analyzed the development of apoplastic barriers under abiotic stresses. The number of articles published in this field increased between 2011 and 2019. The advancements in molecular biology have led to significant progress in understanding the molecular mechanisms regulating apoplastic barriers. However, the number of publications decreased in 2022 compared with 2021, possibly due to coronavirus disease 2019 [20]. Nonetheless, more than 50 publications were reported in the field annually from 2020 to 2023, indicating sustained high interest in the field. Detailed research has been conducted on the formation mechanisms of apoplastic barriers and their response to suboptimal plant growth environments during this period. Specifically, the regulatory network for CS formation in the exodermis of rice has been reported, providing essential insights into the regulatory mechanism of multilayer CS formation [21].

The top 11 publication sources with the maximum number of publications are listed in Table 1, with the highest number of publications (39) in the *Journal of Experimental Botany*; the second and third most published journals were *Frontiers in Plant Science* and *Plant Physiology*, respectively.

**Figure 2:**
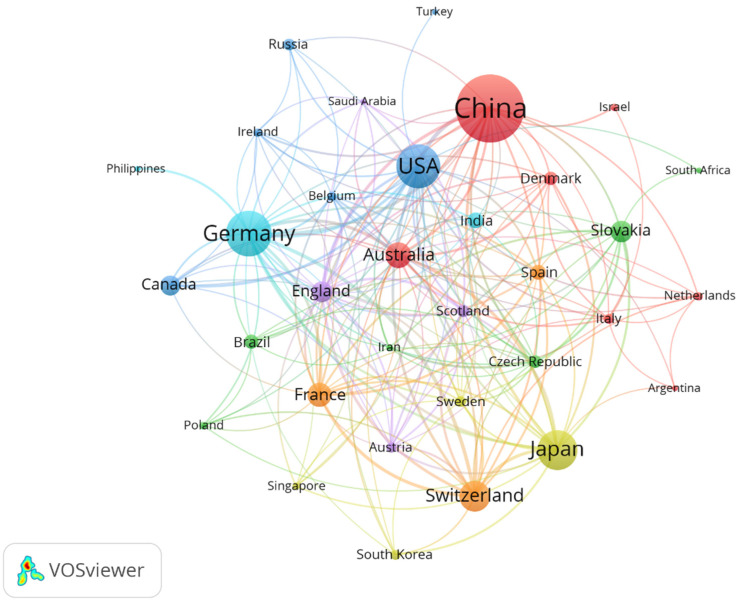
Collaborative network of countries on apoplastic barriers from 2003 to 2023.

The top 10 countries in terms of the number of publications in this field were China, Germany, the United States, Japan, Switzerland, Australia, France, Slovakia, England, and Canada (Table 2). More than 50% of the publications were from China (27.73%), Germany (15.89%), and the United States (14.95%). The line width connecting the two countries is an indication of the connection between them. The more comprehensive the line, the more the research collaborations between the two countries [22]. As shown in Figure 2, Germany, China, and the United States are the countries with more international cooperation in this field, respectively. This may be due to their geographical location and academic level, as some studies have found these factors to be the main considerations for researchers seeking international collaborations [23].

**Figure 3:**
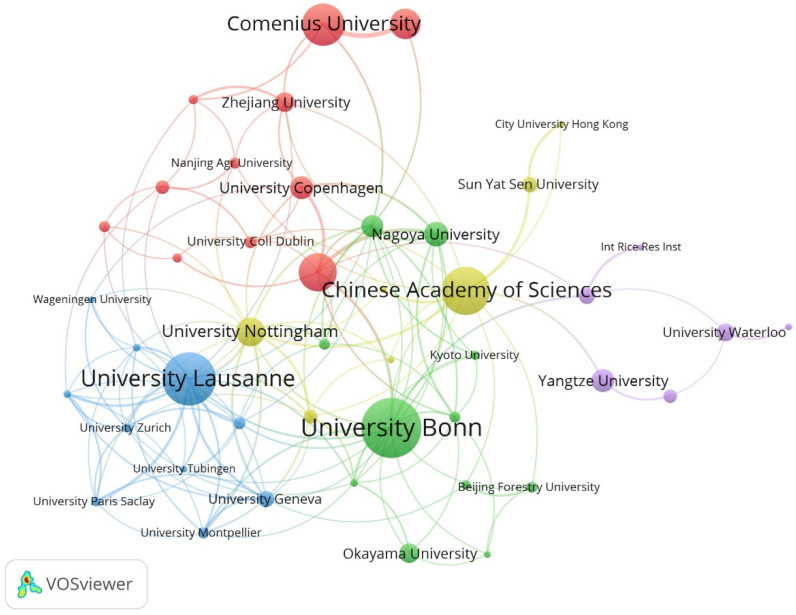
Collaborative network of institutions on apoplastic barriers from 2003 to 2023.

**Figure 4:**
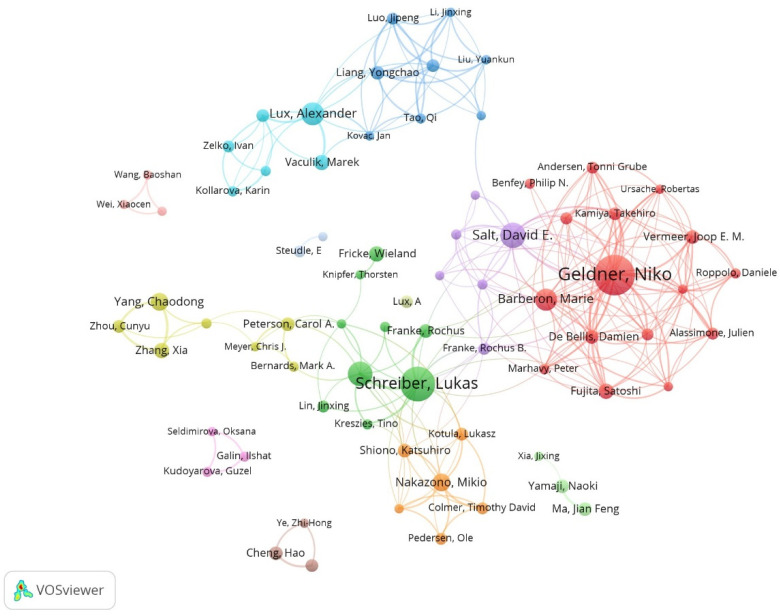
Collaborative network of authors on apoplastic barriers from 2003 to 2023.

The University of Bonn had the maximum number of publications, followed by the University of Lausanne and the Chinese Academy of Sciences (Table 3). At the same time, these institutions have relatively rich collaborative networks, suggesting their frequent collaboration on a global scale (Figure 3). Niko Geldner, Lukas Schreiber, and Marie Barberon are three authors with a maximum number of publications (more than 15 publications each) (Table 4). Similarly, these scholars collaborated more with other researchers, likely due to their stronger academic reputation and higher authority (Figure 4). Overall, this information may assist scholars in the field in finding authoritative collaborators and promoting scholarly exchange.

### 3.2. Highly Cited Publications

The 27 highly cited publications during 2003–2023 were published in 17 journals (Table 5), 4 of which were published in *Plant Cell*. The most cited publication was in *Nature* by Jianfeng Ma [24]. The highly cited publications were mainly divided into four categories: (1) genes, proteins, enzymes, and hormones regulating the formation of apoplastic barriers; (2) the influence of adversity stress on apoplastic barriers; (3) the chemical components of apoplastic barriers; (4) the evaluations of research progress on apoplastic barriers.

More than half of the highly cited publications were from the United States (33.33%) and Switzerland (25.92%). Researchers in both the United States and Switzerland are interested in the molecular mechanisms of apoplastic barrier formation in plant roots, and extensive collaborative research has been conducted in this area. For example, the two countries have collaborated in the discovery of the *SCHENGEN3*/*GASSHO1* (*SGN3*/*GSO1*) receptor-like kinase, enhanced suberin1 (*ESB1*), and Casparian strip domain proteins (*CASP*s), which are essential in regulating the formation of CS. [25,26]. Robertas Ursache [27] found auxin-regulated *GDSL*-lipases driving root suberin plasticity. The transcription factor *MYB36* is essential for CS formation, which directly and positively regulates the expression of the CS genes *CASP1*, *PER64*, and *ESB1* [28]. Moreover, a group of MYB transcription factors (*MYB41*, *MYB53*, *MYB92*, and *MYB9*) have been found to modulate the formation of endoderm suberin [29].

Highly cited publications from Australia, Germany, and Canada have focused on the response of plant root apoplastic barriers to abiotic stress. Research has shown that salt stress leads to the development of apoplastic barriers in the roots towards the root tip. However, this response varies among various plant species. The apoplastic barriers of the endodermis have received more attention than those of the exodermis because fewer plants have multilayered apoplastic barriers [30]. In addition, flooding causes oxygen deprivation in the soil, and the organic acids produced by anaerobic microorganisms promote the formation of CS/SL, thereby reducing radial oxygen loss [31]. Nutrients, especially nitrogen, significantly limit crop productivity. Nitrogen levels and nitrogen forms have complex effects on the formation of CS, but the relationship between nitrogen availability and CS formation remains largely unknown [32].

Currently, numerous researchers have thoroughly investigated these aspects. For example, in terms of the molecular mechanism of CS formation under abiotic stress, *SbCASP-LP1C1* improved salt tolerance of sweet sorghum (*Sorghum bicolor*) by enhancing the root apoplastic barriers and blocking the transport of sodium ions to the shoot [33]. Many recent studies have focused on the plasticity of root apoplastic barriers in different ecotypes of plants. In *S. alfredii*, the development of apoplastic barriers was later in ecotypes with high cadmium accumulation than in ecotypes with low cadmium accumulation, regardless of cadmium content [34]. Liu [35] also concluded that drought-tolerant varieties of *Elymus sibiricus* showed less apoplastic bypass flow of water and solutes than drought-sensitive ecotypes, as determined using a hydraulic conductivity measurement system and an apoplastic fluorescent tracer.

### 3.3. Research Directions

**Figure 5:**
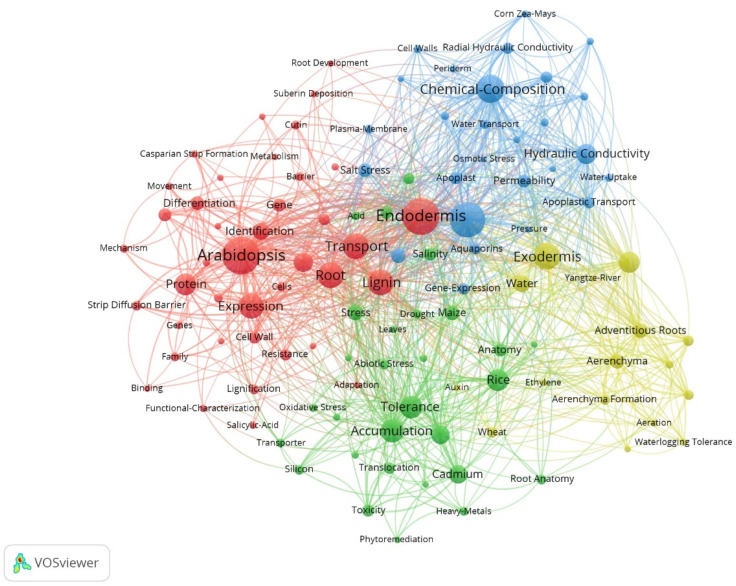
Keyword co-occurrence network in the study of apoplastic barriers.

We used VOSviewer to generate a keyword co-occurrence network to reveal clustering relationships and co-occurrence states further (Figure 5). Four clusters were identified using VOS viewer, namely red, blue, green, and yellow clusters, that corresponded to the different research topics, respectively. The keyword with the highest frequency was *Arabidopsis*. *A. thalianahas* has many advantages in botanical research as a model plant, and it is also the most studied plant in the field.

The red cluster (Figure 5) is mainly related to the formation of apoplastic barriers, and the most frequent keyword is *Arabidopsis*. Other high-frequency keywords are Endodermis, Expression, Transport, Lignin, Root, and Protein. At the molecular level, transcriptomics is widely used to identify regulatory genes for apoplastic barriers. For example, three distinct developmental regions (undifferentiated, developing, and mature) were identified based on CS and SL staining in the root cross-sections of sweet sorghum; the sequencing of RNA extracted from these distinct sections identified essential genes participating in the differentiation of the apoplastic barriers [36]. Propidium iodide (PI) is now widely used as a tracer of the apoplastic pathway to estimate the inhibitory function of CS in testing the integrity and barrier capacity of apoplastic barriers. Fluorescein diacetate (FDA) only becomes fluorescent after its uptake into living cells via the mobility through the plasmodesmata and is used as an effective cell-to-cell tracer [37]. ClearSee is a plant tissue transparency agent that can be combined with classic dyes for lignin and suberin to achieve simple and practical observation of apoplastic barriers. This method is more intuitive and accurate than traditional sectioning [38]. Transmission electron microscopy application allows a more specific quantification of CS thickness for root samples with insignificant fluorescence differences [39].

The blue cluster (Figure 5) is mainly related to the chemical composition of apoplastic barriers, and the most frequent keyword is Suberin, followed by Chemical-Composition. Keywords such as Permeability, Hydraulic Conductivity, and Water Transport also appear frequently. The color difference in phloroglucinol staining can determine the lignin content, and the content of each monomer in suberin should be determined by gas chromatography-mass spectrometry (GC-MS). Aliphatic suberin is generally considered the main barrier to water transport due to its high hydrophobicity, whereas aromatic suberin mainly forms a barrier to the penetration of solutes and pathogens. In maize, high nitrate inhibited the suberization of roots. Also, the content of aliphatic suberin decreased with the increase in NO_3_^−^ concentration, whereas no significant difference in the content of aromatic suberin [40]. Osmotic stress increased the content of aliphatic suberin of mature SL in barley (*Hordeum vulgare*), but there was no difference was found in the elongation zone [41].

The yellow cluster (Figure 5) is mainly associated with water adaptation, with the most frequent keyword being Exodermis, followed by Radial Oxygen Loss. The keywords for this cluster are related to wetlands and aquatic plants, such as Aerenchyma, Waterlogging Tolerance, and Yangtze River. Water adaptation is essential for plants growing in riparian, wetland, and mangrove zones because these plants must adapt to frequently changing soil water content. In addition, these plants often face other soil stresses or water pollution. The CS gap at the junction of the main and lateral roots is an essential pathway for microplastic influx in the root system of the aquatic plant *Eichhornia crassipes*, disrupting the integrity of CS and promoting the migration of microplastics into the vascular system [42].

The green cluster (Figure 5) is mainly related to ion uptake and accumulation. Accumulation and Tolerance are the most frequently occurring keywords, followed by Cadmium, Rice, Responses, and Stress. Heavy metal ions pose a significant threat to plant growth and development and have the potential to affect human health through the food chain. Apoplastic barriers can reduce the radial transport of Cd into the rice root system, thereby decreasing the Cd content of the seeds [43]. Promoting the formation of apoplastic barriers in plant roots can be a promising approach for reducing heavy metal accumulation in crops. The deposition of apoplastic barriers increases resistance to apoplastic flow under salt stress, leading to efficient salt exclusion at the roots, thereby reducing Na^+^ accumulation and affecting ion homeostasis [44]. On the contrary, the effect of nutrient-deficient environments on root apoplastic barriers has received much attention; for example, low calcium-induced delay in the development of root apoplastic barriers enhances Cd uptake and accumulation in *S. alfredi* [45]. This suggests that adequate nutritional conditions are essential for plants to form apoplastic barriers and resist abiotic stresses.

### 3.4. Research Hotspots

**Figure 6:**
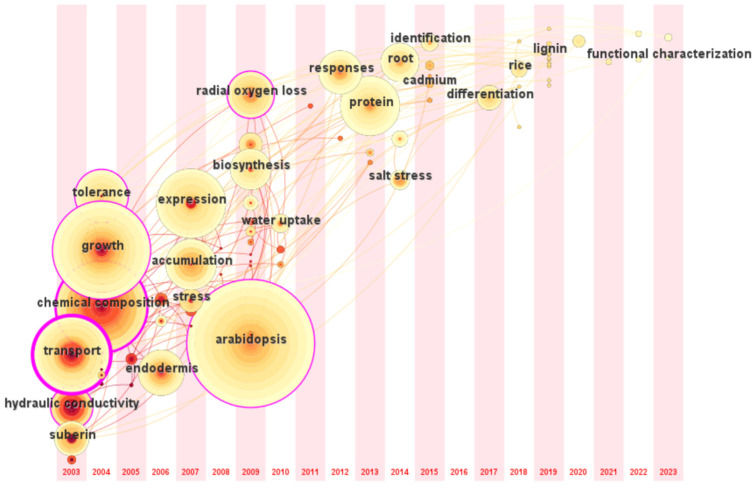
Time zone map of the research on apoplastic barriers. The position of the circle is the time of the first appearance of the keyword; the larger circle indicates that the keyword appears more often.

The research trend in this field based on the time zone map of CiteSpace during 2003–2023 is shown in Figure 6. The earliest and most researched plant in this field is *A. thaliana*, followed by an increasing number of studies on maize, rice, and other crops. At the same time, research interest has increased in wild plants with higher resilience. *Opisthopappus taihangensis*, a wild relative of Chrysanthemum, has upregulated suberin-related genes under drought stress and rapid SL deposition to maintain plant water [46]. Current research focuses on linking the growth characteristics of specific plants to practical problems in crop production. For example, salt-tolerant forages are the primary targets for mining the genes regulating apoplastic barriers under salt stress. At the same time, crops pay more attention to the transport and accumulation of ions from roots to grains. In addition, barley and sweet sorghum have strong resistance to various abiotic stresses; it has received extensive attention and can help improve our understanding of the response of root apoplastic barriers to stress.

Adverse environmental conditions, including abiotic and biotic stresses, can affect plants’ growth. In terms of plant stress physiology, the research hotspot has also gradually shifted from the response of CS/SL to stress to the regulation of the formation of CS/SL to improve plant stress tolerance. Genetic engineering is a feasible technology for achieving this goal. A dirigent protein (called *ZmESBL*) localized to the CS domain has been identified in maize, the CS of *ZmESBL* knockout was less functional than that of the wild-type plants [39]. The overexpression of *ZmLAC3* promoted the formation of CS in maize, and the contents of K, Mn, and Cu in ear leaves of *ZmLAC3*-overexpressing transgenic maize in the field were significantly higher than those in wild type [47]. Nutrient addition is also an effective way to promote the formation of CS/SL to resist stress [48]. The addition of ammonium nitrogen can promote the formation of CS in rice roots, but the addition of excessive or insufficient nitrogen concentration leads to a significant decrease in hydraulic conductivity and solute permeability [49]. Also, some studies have demonstrated that the exogenous addition of selenite can promote the formation of CS and reduce the inflow of cadmium from rice roots [50]. In addition, microorganisms can also induce CS formation [51]. Inoculation with plant growth-promoting rhizobacteria promoted the formation of wheat CS, increasing potassium absorption and hydraulic conductivity [52]. Some scholars also found that arbuscular mycorrhizal fungi promoted the formation of CS in maize roots, regardless of drought stress [53].

The three pathways of radial transport in the root system are inseparable. A better understanding of water and mineral uptake in plant roots can be achieved by focusing on both the apoplastic and cell-cell pathways. Osmotic stress promoted the formation of apoplastic barriers in barley. Still, it barely affected the expression of water channel-related genes, suggesting that osmotic stress reduces water return from the root system to the medium by decreasing water loss in the apoplastic pathway. In contrast, the cell-cell pathway remained unchanged [41]. Salt stress reduces the hydraulic conductivity of the root system by reducing water flow along the cell-cell pathway. The reduction in this conductivity did not require added hydraulic resistance through apoplastic barriers at the endodermis [54]. However, the relationship between apoplastic barriers and mineral element channel proteins is poorly understood [55]. Understanding both the apoplastic barriers and the transporters can help breed crops with high nutrient-use efficiency and yield in the future.

## 4. Conclusions and Outlook

This study provided a comprehensive and visual overview of publications on apoplastic barriers in plant roots based on data from the Web of Science from 2003 to 2023. The number of publications per year in this field has increased significantly since 2011. However, this number has decreased since 2020, but it has stabilized at more than 50. In terms of international cooperation, China is the country with the largest number of publications and maximum cooperation (Figure 2). In the highly cited publications, the molecular mechanism of the formation of apoplastic barriers in plant roots has received maximum attention, which is also the area of the closest international cooperation at present. Meanwhile, the effect of abiotic stress on root apoplastic barriers is also one of the research hotspots, but international cooperation in this field is not close. Based on the analysis of research hotspots and core evolutionary pathways, the directions for future in-depth research are proposed in the light of related studies and reviews. The aim is to provide new theoretical perspectives for the exploration of plant root apoplastic barriers today.

(1) Physiological functions of apoplastic barriers in plant roots. CS and SL are the primary regulators of substance uptake and transport in the apoplastic pathway. Current research on the function of the apoplastic barriers has focused on ionic and water stresses, especially Na^+^ and Cd^2+^. However, the effects of organic pollutants, such as microplastics, nanoplastics, and pesticides, have been neglected. These environmental pollutants negatively affect plant growth and may cause harm to humans throughout the food chain. Future studies should further investigate the role of apoplastic barriers in blocking the transport of harmful substances under complex stresses and the effects of these stresses on the formation of apoplastic barriers.
(2) Differences in the formation of apoplastic barriers with different root systems. Most of the current research on apoplastic barrier formation has focused on primary and seminal roots, especially root tips, while apoplastic barriers in lateral roots have been largely ignored. The relationship between root structure and function has significant variations due to the differences in root structure and the absorption and transport capacity of roots at different root levels. Moreover, the stem nodes also give rise to root systems for plants with rhizomes or stolons. Whether differences exist in the development of apoplastic barriers between root systems at different locations in the plant remains unexplored. In summary, we should gain a more in-depth understanding of the differences in apoplastic barriers formed by the root system at different levels and locations to fully understand the plasticity of plant apoplastic barriers.
(3) Methods to promote apoplastic barrier formation. Exogenously added plant growth regulators (e.g., nano-silver, activated carbon, and novel phytohormones) have significant potential to improve plant productivity. A large number of studies have demonstrated the beneficial effects of these growth regulators on plants. However, little is known about their effects on apoplastic barriers. Cerium oxide nanoparticles retarded the formation of apoplastic barriers in the root system of *Brassica napus*, enabling the transport of more Na^+^ to the aboveground parts and reducing Na^+^ accumulation in the roots [56]. Rhizosphere microorganisms can promote the formation of apoplastic barriers. Exploring the role of different exogenous additives and rhizosphere microorganisms can provide methodological and theoretical support for improving crop resistance and varieties in agriculture.
(4) Application of molecular biology techniques. The study of apoplastic barriers has entered an entirely new phase with the development of molecular biology techniques such as genomics (macrogenomics, transcriptomics, proteomics, and metabolomics) and transgenics. Molecular biological techniques enable the exploration of critical genes and proteins regulating apoplastic barrier formation. On the contrary, the overexpression of related genes through transgenic technology can improve stress resistance and the quality of crops. The study of apoplastic barriers will inevitably use the latest research techniques to explore the intricate molecular mechanisms governing the developmental patterns of apoplastic barriers, as well as their intricate relationships with plant growth and resistance to stresses.

## Figures and Tables

**Table 1:** Top 11 journals that published articles about apoplastic barriers from 2003 to 2023.

Rank	Publication Sources	Number of Articles	Percentage (%)
1	Journal of Experimental Botany	39	6.07
2	Frontiers in plant science	32	4.98
3	Plant physiology	31	4.83
4	New phytologist	23	3.58
5	Annals of botany	22	3.43
6	Plant, Cell & Environment	15	2.34
6	Plant Journal	15	2.34
8	Proceedings of the National Academy of Sciences of the United States of America	14	2.18
9	Plant and soi	13	2.02
10	Environmental and Experimental Botany	11	1.71
10	Plant cell	11	1.71

**Table 2:** Top 10 countries that published articles about apoplastic barriers from 2003 to 2023.

Rank	Country	Number of Articles	Percentage (%)
1	China	178	27.73
2	Germany	102	15.89
3	The United States	96	14.95
4	Japan	84	13.08
5	Switzerland	58	9.03
6	Australia	47	7.32
7	France	41	6.39
8	Slovakia	37	5.77
9	England	35	5.45
10	Canada	32	4.98

**Table 3:** Top 10 institutions that published articles about apoplastic barriers from 2003 to 2023.

Rank	Institution	Number of Articles	Percentage (%)
1	University of Bonn	41	6.39
2	Chinese Academy of Sciences	38	5.92
3	University of Lausanne	36	5.61
4	Comenius University in Bratislava	30	4.67
5	National Research Institute for Agriculture, Food and Environment	27	4.21
6	University of Western Australia	25	3.89
7	Centre National de la Recherche Scientifique	24	3.74
8	Slovak Academy of Sciences	18	2.80
8	University of Nottingham	18	2.80
10	Nagoya University	15	2.34

**Table 4:** Top 11 authors that published articles about apoplastic barriers from 2003 to 2023.

Rank	Author	Number of Articles	Percentage (%)
1	Niko Geldner	39	6.07
2	Lukas Schreiber	23	3.58
3	Marie Barberon	17	2.65
4	Kosala Ranathunge	15	2.34
4	David E. Salt	15	2.34
6	Alexander Lux	11	1.71
7	Chaodong Yang	9	1.40
8	Carol A. Peterson	8	1.25
8	Mikio Nakazono	8	1.25
8	Rochus Benni Franke	8	1.25
8	Hao Cheng	8	1.25

**Table 5:** Analysis of the journal sources of the highly cited publications on apoplastic barriers from 2003 to 2023.

Rank	Publication Sources	Number of Publication	Percentage (%)
1	Plant cell	4	14.81
2	Proceedings of the National Academy of Sciences of the United States of America	3	11.11
2	New phytologist	3	11.11
3	Science	2	7.40
3	Journal of Experimental Botany	2	7.40
3	Annual review of phytopathology	2	7.40
4	Nature Plants	1	3.70
4	Nature Communications	1	3.70
4	Plant Science	1	3.70
4	Phytochemistry	1	3.70
4	Plants-basel	1	3.70
4	Nanotoxicology	1	3.70
4	Current Opinion in Plant Biology	1	3.70
4	Plant Cell Reports	1	3.70
4	Plant Physiology	1	3.70
4	Frontiers in plant science	1	3.70
4	Plant Communications	1	3.70

## Data Availability

Data are contained within the article.
